# Setting up a wide panel of patient-derived tumor xenografts of non–small cell lung cancer by improving the preanalytical steps

**DOI:** 10.1002/cam4.357

**Published:** 2014-12-03

**Authors:** Marius Ilie, Manoel Nunes, Lydia Blot, Véronique Hofman, Elodie Long-Mira, Catherine Butori, Eric Selva, Ana Merino-Trigo, Nicolas Vénissac, Jérôme Mouroux, Patricia Vrignaud, Paul Hofman

**Affiliations:** 1 Laboratory of Clinical and Experimental Pathology, Louis Pasteur HospitalNice, France; 2 IRCAN Team 3, Inserm U1081/UMR CNRS 7284, Faculty of Medicine of Nice, University of Nice Sophia AntipolisNice, France; 3 Faculty of Medicine, University of Nice Sophia-AntipolisNice, France; 4 Hospital Related Biobank BB-0033-00025, Louis Pasteur HospitalNice, France; 5 Oncology Department, Sanofi LaboratoriesVitry-sur-Seine, France; 6 SCP Biologics Department, Sanofi LaboratoriesVitry-sur-Seine, France; 7 Department of Thoracic Surgery, Louis Pasteur HospitalNice, France

**Keywords:** Molecular pathology, NSCLC, PDX, preanalytical

## Abstract

With the ongoing need to improve therapy for non–small cell lung cancer (NSCLC) there has been increasing interest in developing reliable preclinical models to test novel therapeutics. Patient-derived tumor xenografts (PDX) are considered to be interesting candidates. However, the establishment of such model systems requires highly specialized research facilities and introduces logistic challenges. We aimed to establish an extensive well-characterized panel of NSCLC xenograft models in the context of a long-distance research network after careful control of the preanalytical steps. One hundred fresh surgically resected NSCLC specimens were shipped in survival medium at room temperature from a hospital-integrated biobank to animal facilities. Within 24 h post-surgery, tumor fragments were subcutaneously xenografted into immunodeficient mice. PDX characterization was performed by histopathological, immunohistochemical, aCGH and next-generation sequencing approaches. For this model system, the tumor take rate was 35%, with higher rates for squamous carcinoma (60%) than for adenocarcinoma (13%). Patients for whom PDX tumors were obtained had a significantly shorter disease-free survival (DFS) compared to patients for whom no PDX tumors (*P *=* *0.039) were obtained. We established a large panel of PDX NSCLC models with a high frequency of mutations (29%) in *EGFR*, *KRAS, NRAS*, *MEK1*, *BRAF*, *PTEN*, and *PI3KCA* genes and with gene amplification (20%) of *c-MET* and *FGFR1*. This new patient-derived NSCLC xenograft collection, established regardless of the considerable time required and the distance between the clinic and the animal facilities, recapitulated the histopathology and molecular diversity of NSCLC and provides stable and reliable preclinical models for human lung cancer research.

## Introduction

Despite some recent improvements in therapies targeting specific genomic alterations, lung cancer remains the first leading cause of cancer deaths worldwide 1. Continued effort in classification of lung cancer based on gene expression profiling and genomic sequencing have revealed underlying complexity and molecular heterogeneity within the disease, which continues to be a challenge for therapeutic intervention 2,3. To successfully identify and translate new treatment regimens to the clinic, it is critical that robust preclinical models that faithfully model human cancers recapitulate this complexity.

Patient-derived tumor xenografts (PDX), established by implanting fresh patient tumor fragments into immunodeficient mice, subcutaneously or orthotopically, are reported to be more relevant to the clinical context than cell-based tumor xenografts 4–7. This model system involves serial propagation in mice of tissue explants and avoids cell culture on Petri dishes. The use of standardized procedures for the assessment of the therapeutic efficacy of different drugs using PDXs allows rapid assessment of combined therapies on a relatively large set of tumors 8. To date, only a few studies developed PDX models for NSCLC 4,7–10. Recent evidence suggests that PDX models can maintain certain pathological and molecular features of the patient's tumor 11. A limited number of studies have extensively characterized these PDX models at the histological, molecular, and pharmacological levels to ensure that they truly represent the diversity of the clinical situation 4,6. Some studies provided evidence of a potential correlation of responses of anti-EGFR therapies within PDX models harboring *EGFR* activating mutations 4,6. However, the number of primary NSCLC tumors included and the rate of engrafted tumors were limited. To our knowledge, surgical fragments must be processed rapidly and there is often a limited amount of the original tumor material, which hinders extensive standardized translation into clinical facilities worldwide.

Here, through a consortium effort of hospitals, academic groups, and pharmaceutical company, we have developed a large collection of NSCLC models directly derived from tumor samples collected during patient surgery within a context of a long-distance research network. Fresh patient tumor fragments were maintained in survival medium and then shipped at room temperature to a distant site with animal facilities located at around 1000 km/620 miles away. Starting from 100 surgically resected NSCLC specimens, we have established 35 transplantable PDXs. We have characterized these xenograft models at the histological and immunohistochemical level as well as the molecular level by using next-generation sequencing (NGS) and array (comparative genomic hybridization, CGH) analysis to ensure that they represent the diversity of the clinical situation. All the characteristics of the models and clinical patient history are being loaded into an internal database for efficient use in terms of target validation, biomarker discovery, and preclinical evaluation of new agents.

In conclusion, the establishment of this collection holds great promise not only to further develop personalized approaches for the treatment of NSCLC patients but also for wide implementation in clinical trials of these xenograft models located at a distance from the collecting area.

## Materials and Methods

### Patient recruitment

One hundred tumor specimens were obtained at initial surgery from primary diagnosed NSCLC patients from the Hospital related Biobank of the Pasteur Hospital (BB-0033-00025, Nice, France). Only six patients received a neoadjuvant chemotherapy. Written informed consent was obtained from each patient and the study was approved by the hospital ethics committee. To protect the manipulators, only patients seronegative for HIV1&2 and HBV and HCV were included in this study. Tumor histology was assessed by four pathologists (M. I., V. H., E. L., and C. B.). Immediately after surgery (on average 1 h after resection), two or more fragments were fixed in 10% neutral buffered formalin (Diapath, Martinengo, Italy) and paraffin-embedded (Thermo Fisher Scientific, Villebon-sur-Yvette, France) for histological analysis, five fragments were snap frozen in liquid nitrogen, then stored at −80°C for molecular characterization, and several small fragments (from 0.5 to 1 cm^3^), depending on the tumor size and area of necrosis, were transferred in culture medium for engraftment, which was carried out within 24 h after resection. The time of warm ischemia was recorded by the surgeon for each specimen and the time of cold ischemia was recorded using radio-frequency identification (RFID) technology 12. A similar process of sample conservation was applied to tumor fragments collected from mice.

### Animals

All experimental procedures were approved by the Sanofi, Animal Care and Use committee. CD1 nude and CB17-SCID female mice were obtained from Charles River France. The mice (at least 7-week old at the start of engraftment) had free access to food and water.

### Tumor engraftment and PDX maintenance

Fresh patient tumor specimens were maintained in AQIX® medium (AQIX Ltd., The London Bioscience Innovation Centre, London, UK) at room temperature for shipment from the Nice (France) Hospital Biobank to the Sanofi animal facilities in Vitry-sur-Seine (France) within 24 h post-surgery. The estimated distance between the facilities is about 1000 km/620 miles. The nonnecrotic tumor tissue was cut into 5 × 5 mm fragments (without dissociation to maintain the tissue integrity) and subcutaneously (SC) engrafted into five female SCID mice using a 12-gauge trocar. The mice were housed in isolators for 7 weeks to control if their sanitary status was specific and opportunist pathogen-free (SOPF). The exponentially growing tumors were passaged SC to other mice and maintained for a maximum of 10 passages in mice. The tumor growth in CD-1 nude and SCID mice was evaluated to select the best strain for tumor maintenance and cryopreservation. Using Recovery™ Cell Culture Freezing Medium (Gibco®, Grand Canyon, NY), the cryopreservation was performed at passages 1–5 (up to 8 for some slow-growing PDX), to generate a tumor bank of frozen tissue, which allowed the study of patient-derived NSCLC xenografts (PDX) at only low passages. Tumor growth post thawing was checked at a passage close to the one used for PDX characterization. Depending on the tumor growth the passage of characterization was usually between passage 3 or 5, but in some cases it was extended up to passage 8. The tumor take rate was calculated taking into account the established tumor models, that is, tumor models successfully passaged at least twice and validated as a human tumor (no contamination by a murine or human lymphoma).

### PDX tumor growth characterization

Tumor volumes were estimated by two-dimensional measurement using the formula: tumor volume (mm^3^) = [length (mm) − width^2^ (mm^2^)]/2. The tumor doubling time (Td) in days was estimated from the log linear tumor growth during the exponential phase (range, 100–1000 mm^3^).

### Histopathological, immunohistochemical, and FISH characterization

Examination of the PDX morphology was conducted on slides obtained from serial 5-*μ*m-thick sections cut from each formol-fixed paraffin-embedded PDX blocks at early and late passages and processed for hematoxylin–eosin–safran (HES) staining. A mouse monoclonal anti-TTF1 antibody (clone 8G7G3/1; Ventana Medical Systems, Roche Group, Tucson, AZ), mouse monoclonal anti-p63 antibody (clone 4A4; Ventana Medical Systems) and rabbit monoclonal anti-c-MET antibody (Ventana) were used for immunohistochemistry. Control sections were processed in parallel with rabbit or mouse nonimmune IgG (Dako, Carpinteria, CA) used at the same concentrations as the primary antibodies. A dual color FISH analysis was performed using a *c-MET* specific FISH probe (Vysis MET SpectrumRed FISH Probe Kit; 7q31.2) and chromosome 7 centromeric probe CEP (Vysis CEP7 SpectrumGreen probe; Abbott Molecular Inc., Des Plaines, IL) as previously described 13. For the *FGFR1* FISH assay, 4-*μ*m tumor tissue sections were hybridized overnight with the ZytoLight SPEC FGFR1/CEN 8 Dual Color Probe (ZytoVision, Bremerhaven, Germany), as previously described 14. Target gene and CEP signals were observed using a fluorescence microscope (Eclipse 80i; Nikon, Champigny-sur-Marne, France) equipped with the appropriate filters. Enumeration of the *c-MET* or *FGFR1* genes and chromosome 7 or 8 were conducted by microscopic examination of at least 60 tumor nuclei, which yielded a ratio of *c-MET/CEP7* or *FGFR1/CEP8*. Tumors with a ratio ≥2 or presence of ≥10% gene cluster were defined as amplification 6,14.

### DNA sequencing of patient samples and PDX models

NGS and mutation calling of PDX models were performed at the Beijing Genomics Institute (BGI, Beijing, China). Library preparation was performed using exome capture Agilent SureSelect All Exon 50M. Libraries were sequenced using the Illumina HiSeq platform. The normal human genomic DNA used in these experiments was purchased from the Coriell Institute for Medical Research (Camden, NJ). DNA samples from each PDX were sequenced and compared with their constitutive DNA from patients as a control.

### Array CGH analysis on PDX models

Evaluation of the genome-wide, gene copy number was performed using the 400k CGH Agilent technology array. Oligonucleotide aCGH processing was performed as detailed in the manufacturer's protocol (version 6.2 October 2009; http://www.agilent.com). The microarray required 600 ng of genomic DNA from the reference sample and from the experimental sample. The array was scanned with an Agilent DNA Microarray Scanner (G2565CA). Data were extracted from scanned images and normalized using Feature Extraction software (v10.7.3.1; Agilent). In all experiments, sex-matched DNA from a human well-characterized normal female (NA12878) or one well-characterized normal male (NA10858) was used as reference DNA. The normal human genomic DNA used in these experiments was purchased from the Coriell Institute for Medical Research (Camden, NJ). The log_2_ ratio and segmentation were generated using Array Studiosoftware. Array Studio, Array Viewer, Array Server, and all other Omicsoft products or service names are registered trademarks or trademarks of Omicsoft Corporation (Research Triangle Park, NC). All genomic coordinates were established on the UCSC human genome build hg19 15.

Gene amplification or high-gain is defined by a copy number (CN) ≥ 5 or log_2_ ratio ≥ 1.32) and gene gain or low gain by (2.5 ≤ copy number (CN) < 5 or 0.32 ≤ log_2_ ratio < 1.32).

### Statistical analysis

To identify clinical parameters that contribute to the success of establishment of PDX, a logistic regression analysis was used to assess the association of the success rate of establishment with clinic-pathological parameters and DFS evaluation. A patient tissue that was successfully turned into a xenograft model was defined as 1 and 0 otherwise. *P*-values from univariate models were computed from the log likelihood ratio test. Factors that showed significant results from a univariate analysis were considered in a multivariate analysis to adjust for imbalance of covariates, including sex, age, histology, and pTNM stage. DFS was calculated for patients with documented follow-up of at least 12 months and was defined as time between surgery and relapse or death. DFS percentages were calculated using the Kaplan–Meier method and the survival curves were compared with a log-rank test. Variables of interest were tested in the presence of other clinical factors using a Cox proportional hazards model. Analyses were performed using SPSS 16.0 statistical software (SPSS Inc., Chicago, IL). All statistical tests were two-sided, and *P* < 0.05 indicated statistical significance.

## Results

### Establishment of patient-derived NSCLC xenograft mouse models

One hundred consecutive NSCLC specimens from primary tumors were harvested from patients and subcutaneously engrafted into immunodeficient mice within 24 h post-surgery. The main clinical parameters of the patients are summarized in Table1. Of the 100 engraftments, 35 led to the establishment of PDX models, representing a tumor take rate of 35% (Table1). The tumor take rate was jeopardized by human lymphomagenesis contaminating 13 models. Two PDX models were also discarded due to emergence of murine lymphoma.

**Table 1:** Clinical and pathological characteristics of 100 patients and their tumors according to the engraftibility

Patient characteristics *(n *=* *100)	No PDX (*n* = 65)	PDX (*n* = 35)	*P*-value (univariate)	*P*-value (multivariate)
Age (years)			0.500	0.834
Median (range)	64 (41–87)	69 (55–83)		
Sex			0.008	0.117
Male	39 (60%)	30 (86%)		
Female	26 (40%)	5 (14%)		
Neoadjuvant chemotherapy^1^	2 (3%)	4 (11%)	0.093	0.216
Smoking status			0.086	0.256
Former or current smokers	37 (57%)	26 (74%)		
Never smoked	28 (43%)	9 (26%)		
Histological cell type			<0.001	0.013
Invasive adenocarcinoma	46 (71%)	7 (20%)		
Squamous cell carcinoma	14 (22%)	24 (69%)		
Large cell carcinoma	2 (3%)	2 (6%)		
Sarcomatoid carcinoma	3 (4%)	1 (3%)		
Combined SCLC-SCC	0 (0%)	1 (3%)		
Median tumor size (range) cm	3.2 (1.2–10)	4.5 (2–9.5)	0.500	0.879
Median tumor cell content (range) %	50 (15–90)	50 (10–80)	0.371	0.758
pTNM stage			0.050	0.342
I	31 (48%)	8 (23%)		
II	19 (29%)	16 (46%)		
III/IV	15 (23%)	11 (31%)		
Differentiation grade			0.024	0.362
Well	28 (43%)	7 (20%)		
Moderate	16 (25%)	17 (49%)		
Poor	21 (32%)	11 (31%)		
Mutation status			0.139	0.538
*EGFR* mutation	2 (3%)	1 (3%)		
*KRAS* mutation	11 (17%)	2 (6%)		
15 drivers genomic alterations	17 (26%)	15 (43%)		

TNM, tumor node metastasis; PDX, patient-derived tumor xenografts; SCC, squamous cell carcinomas; SCLC, small cell lung cancer.

1 Neodjuvant chemotherapy: cisplatin–docetaxel regimen.

A prolonged time of warm ischemia significantly caused failure of engraftment (*P *=* *0.033; Fig. S1). In contrast, little impact of the time of warm and cold ischemia together was noted (*P *=* *0.358; Fig. S1).

A logistic regression analysis was used to identify clinical variables associated with a high or low probability of in vivo tumor take (Table1). A multivariate analysis showed that the tumor histological cell type was the only parameter that had a significant impact on the success rate of establishment of PDX. Squamous cell carcinomas (SCC) was much more prone to be tumorigenic in immunocompromised mice (28/47; 60%) compared to adenocarcinomas (ADC; 7/52; 13%). Other factors, including sex, smoking status, neoadjuvant chemotherapy, pathologic grade, and mutation, did not correlate with the success rate; although a univariate analysis showed that sex, smoking status, pTNM stage, and differentiation grade may have an impact when these parameters are considered alone. A biobank was established for all models and the success rate after thawing reached 89%.

### Characteristics and stability of PDX models

**Figure 1:**
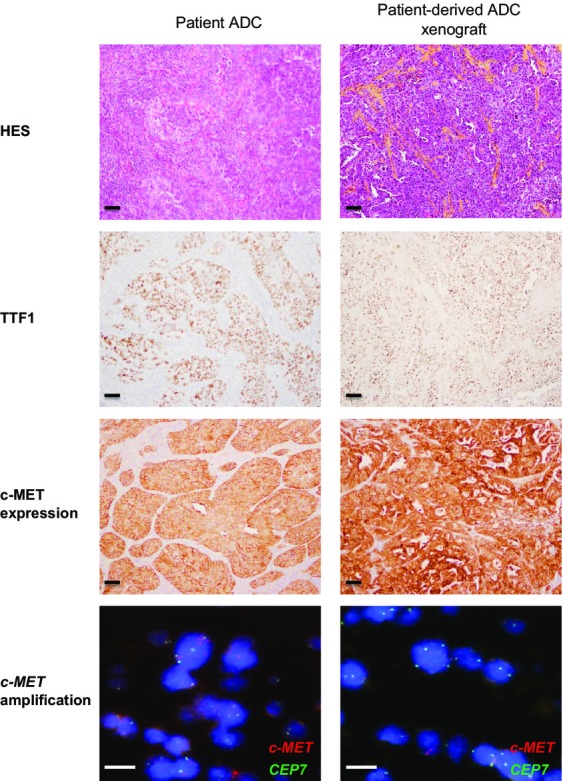
PDX models of lung adenocarcinoma (ADC) specimens recapitulate primary tumor histopathological and phenotype. Hematoxylin–eosin–safran (HES), immunohistochemical (TTF1, c-MET), and *c-MET* FISH analysis of the original patient tumor and xenograft passages for one ADC selected model (LUN-NIC-0084). Scale bar, 10 *μ*m. PDX, patient-derived tumor xenografts.

**Figure 2:**
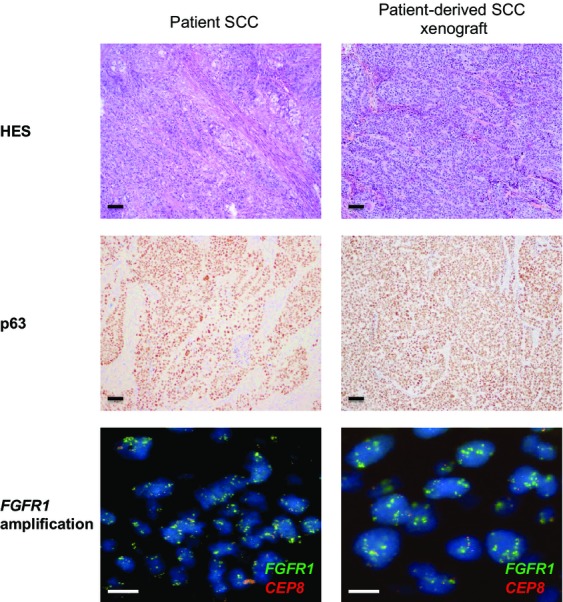
Preservation of the tumor histology and phenotype of a squamous cell carcinoma (SCC) PDX. Hematoxylin–eosin–safran (HES), immunohistochemical (p63), and *FGFR1*FISH analysis of the original patient tumor and xenograft passages for one SCC selected model (LUN-NIC-0007). Scale bar, 10 *μ*m. PDX, patient-derived tumor xenografts.

To evaluate whether stable xenografts retained histological features and if the expression pattern of a number of selected biomarkers was consistent with the tumor of origin, we conducted comparative histopathological and immunohistochemical analyses of outgrowth using clinically relevant biomarkers (e.g., TTF1, p63), and compared the biomarker status with the tumor of origin (Figs.1 and 2).

The PDX NSCLC tissues stained for HES and diagnostic markers (e.g., TTF1 or p63) exhibited a similar morphology and immunophenotype to that of the patient tissues from which the primary model was derived (Figs.1 and 2). The relative state of differentiation is also retained on passaging (two different passages between P6 and P12). PDX were enriched in tumor cells compared with patient samples and the human stroma was finally replaced with a murine stroma during successive passages of the tumors in mice. A fair degree of stroma was retained throughout passaging but did appear to decrease somewhat in comparison to that observed in the primary tumors. Overall, these observations confirm that this PDX collection recapitulates the main histological and immunophenotype profile of corresponding NSCLC.

### Correlation of engraftibility and clinical outcome of NSCLC patients

**Figure 3:**
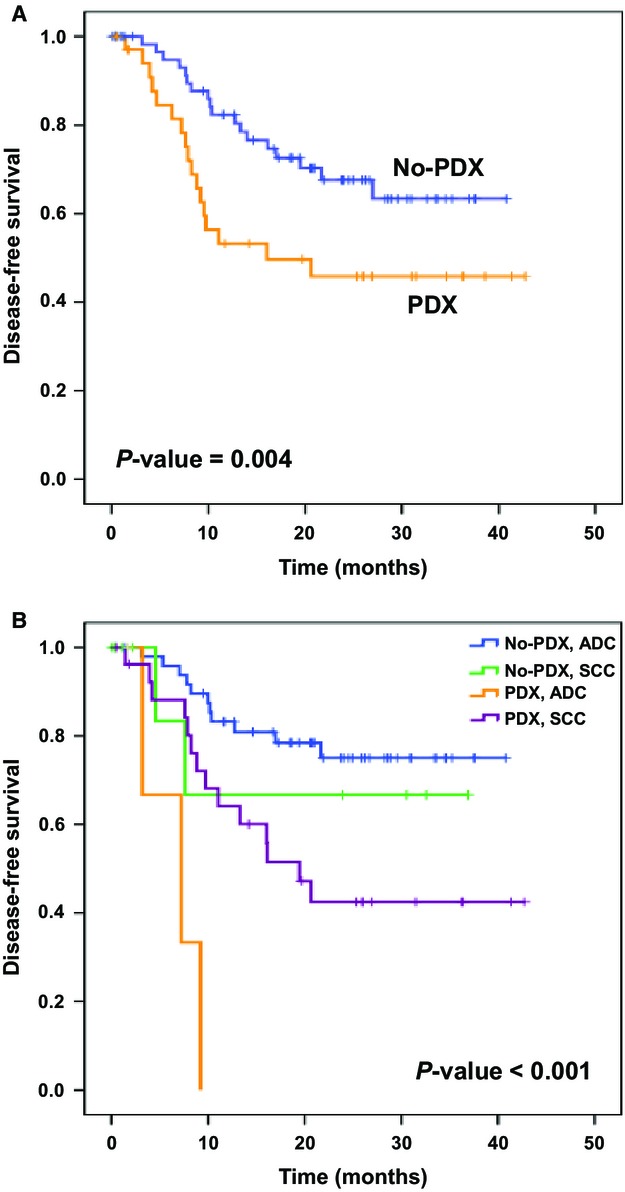
Correlation between engraftibility and the patients' clinical outcome. (A) Kaplan–Meier survival curves for PDX and no PDX patients. (B) DFS according to engraftibility in relationship to tumor histology. DFS, disease-free survival; PDX, patient-derived tumor xenografts.

To determine the impact of engraftment on the clinical outcome of the patients we assessed the DFS of the NSCLC patients included in our study. The median follow-up time was 24 months (range: 3–42.7 months). Thirty-five patients developed clinical recurrence and 17 patients died of lung cancer metastases. Patients for whom tumor engraftment was successful had a significantly shorter DFS than those for whom no PDX was obtained (Fig.3A). Among the patients for whom a PDX was obtained, those with an adenocarcinoma had a worse DFS than those with a SCC (Fig.3B). Conversely, patients with an adenocarcinoma and no PDX had a better DFS than those with a SCC. In the multivariate analysis, in which patient age, sex, histology, and pTNM stage were taken into account, the success of engraftment remained an independent predictor of shorter DFS (Table2).

**Table 2:** Disease-free survival according to the patient and tumor characteristics

Prognostic factor	HR	95% CI	*P-*value
Age	0.751	0.312–1.808	0.524
Sex (M vs. F)	1.142	0.510–2.558	0.746
Histology (ADC vs. other)	0.607	0.241–1.530	0.290
pTNM stage (I + II vs. III + IV)	0.462	0.235–0.910	0.026
Engraftment (yes vs. no)	1.040	1.002–1.079	0.039

M, male; F, female; ADC, adenocarcinoma; TNM, tumor node metastasis.

### Molecular characterization of patient-derived NSCLC xenograft models

**Figure 4:**
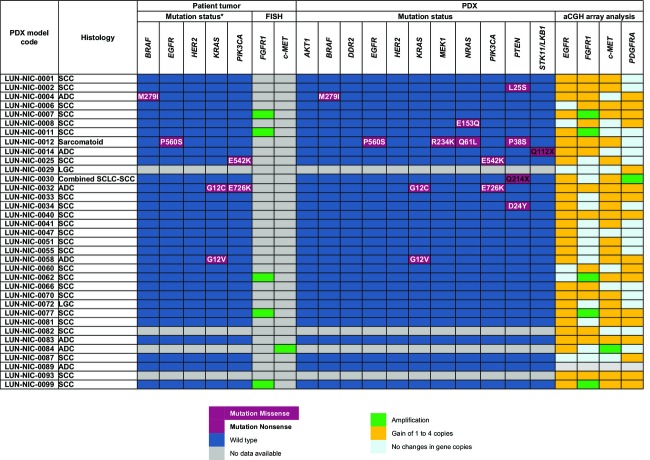
Molecular profile comparison of primary tumors and the 35 PDX NSCLC models using next-generation sequencing and array CGH analyses. *The mutation status in patients' tumor is shown for a panel of genes that are currently of interest in routine practice for NSCLC. Analysis of *FGFR1* and *c-MET* copy number variation of patient samples was performed by FISH assay. aCGH data were generated for PDX samples. PDX, patient-derived tumor xenografts; aCGH, array comparative genomic hybridization.

To investigate the molecular abnormalities displayed by the PDX models we performed genomic profiling by NGS and aCGH analysis. We examined a panel of genes that are of particular interest in current targeted therapies and targeted drug development for NSCLC 16,17. NGS analysis detected mutations in 10 of 35 (29%) PDX models (Fig.4). These included resistant *KRAS* mutations (p.Gly12Cys and p.Gly12Val) in two of seven (29%) adenocarcinoma PDX. A frequency that is consistent with previous reports on the incidence of mutations in this gene in lung adenocarcinoma 18. Additionally, we detected one infrequent *EGFR* mutation in a sarcomatoid PDX and one *BRAF* mutation in an adenocarcinoma model. *PI3KCA* mutations were detected in one SCC and one adenocarcinoma model. *FGFR1* gene amplification was detected in 5 of 24 (21%) SCC models and *c-MET* gene amplification in one (14%) adenocarcinoma model. One sarcomatoid tumor sample harbored four mutations in the *EGFR*, *MEK1*, *NRAS*, and *PTEN* genes, respectively. The overall frequency of gene mutations and amplifications in this collection was similar to the frequency reported in the literature for NSCLC (Fig.4). A similar genomic profile was found in the corresponding primary tumors (Fig.4).

## Discussion

As individualized targeted therapeutic approaches have become more common in NSCLC drug development, models that reflect the patient heterogeneity and faithfully recapitulate the in vivo tumor biology are crucial to improve prediction of clinical efficacy of novel therapies and to reduce the attrition rate in oncology 11. As the recent approval of the *EGFR* and *ALK* inhibitors for the treatment of NSCLC, research has focused on the use of preclinical models and tissue biopsies from patients to examine the mechanisms of responsiveness and resistance to these molecules or to emerging targeted agents 11,19. The establishment of PDX models from NSCLC specimens has been recently investigated as a potential source of therapeutically relevant information and as a supply of biological material 20–24. Most studies report on patient-derived NSCLC xenograft models established from fresh NSCLC samples obtained from patients undergoing surgical resection and immediately implanted into mice, which show a tumor take rate ranging from 25% to 40% 4,7. One group used the technique of directly implanting frozen NSCLC samples from patients into mice and showed a success rate of 32% 6. However, an essential feature of the establishment of PDX models should include the standardization of the collection and shipping at room temperature to a central animal facility.

In a context of a long-distance research network, we have generated 35 patient-derived NSCLC xenograft mouse models by directly grafting fresh patient tumor fragments into SCID mice. The fresh tumor fragments were shipped at room temperature in survival medium and grafted after no more than 24 h post-surgery. For this single-centered collection we did not note any impact on the take rate based on the duration of the cold ischemia. Control of the time of ischemia was carried out using RFID technology Accurate selection of the tumor zones of interest by senior pathologists and the use of the AQIX survival medium contributed to the high rate of engraftment observed in this study. Using this methodology, we achieved an engraftment success rate of 35%, which is within a similar range to that achieved for other systems 6,7. Lymphomagenesis of human origin was observed in 13 models, the primary xenografts being performed in SCID mice, which reduced the final tumor take rate. In switching the strain of mice from SCID to nude mice, this type of contamination no longer occurred 25. In our goal to develop a platform approach, our PDX models were also successfully engrafted on nude mice, known to exhibit metabolic and drug pharmacokinetic/pharmacodynamic profiles closer to humans than SCID mice and are preferable for drug discovery due to their superior tolerability of agents 26. Therefore, these PDX could be used in the mouse for studies into drug efficacy and pharmacokinetics/pharmacodynamics. As it can be very expensive and unpractical to continuously passage PDXs in mice we set up a collection of frozen PDX samples.

The characteristics of the PDXs models were representative of the histological heterogeneity of NSCLC, covering all the major histological subtypes 27. Here, as in other studies 6,7, the primary tumor histology was the major clinical feature to have an impact on the engraftment rate. SCC had a higher success rates in comparison to all other histological subtypes of PDXs. While other patients' characteristics such as age, sex, smoking status, tumor size, tumor cell content, pTNM stage, or differentiation grade, had no impact on the engraftment rates in a multivariate analysis, still the univariate analysis showed that the male sex, higher pTNM stage, and a moderate to poor differentiation grade affected the take rate of PDXs. An interesting and potentially clinically relevant application of these PDX models in NSCLC would be the ability to predict, based on the success of PDX engraftment, those patients who are more likely to relapse after curative surgery, with the added benefit of having an available “Mouse Avatar” for therapeutic testing prior to actual relapse in the patient 11,28. We and others show that patients for whom tumors were able to form xenografts had a significantly shorter DFS, in a multivariate analysis, than patients whose tumors failed to engraft 7. This argues in support of the use of these models in early-stage disease to develop more-effective (and patient-directed) adjuvant chemotherapy 11,23.

The success of targeted therapies in stratifying treatment has underscored the importance of performing mechanistic and functional investigations on NSCLC. Current approaches of personalized medicine have been incorporating NGS technologies for wide genomic profiling of patient tumors to identify novel therapeutic targets. In this study, we report extensive molecular characterization of the 35 NSCLC PDX models using NGS technology and aCGH analysis. The analysis of a panel of genes of particular interest in current targeted therapy and targeted drug development for NSCLC showed that the genomic profile is consistent between the original patient tumor and the corresponding PDX tissue in all the models. When early and relatively late passages of xenografts were compared, no major differences could be observed in terms of alteration in the copy number or the gene mutation profile. This clearly points to the stability of NSCLC xenograft models, as depicted for breast, colon, and pancreatic tumor xenografts 29–31. With respect to the issue of molecular diversity of NSCLC, our novel xenograft models represent useful tools for preclinical evaluation of targeted drug candidates.

In conclusion, we established a large panel of 35 PDX NSCLC models in a context of a long-distance research network. We showed that PDXs maintain a high degree of similarity with the original clinical tumor sample with regard to histology, immunohistochemistry, and the genomic profile. This PDX panel is currently being used in a prospective way to target expression, biomarker discovery, and preclinical evaluation of personalized therapies by the members of the consortium. Our model system, regardless of the time and distance between the location of sampling and engraftment, provides a stable and reliable animal model for human lung cancer research.

## Supporting Information

**Figure S1.** Boxplots for comparison of warm ischemia (A) and the sum of the times of warm and cold (B) ischemia on *Y*-axis with the engraftment of PDTX NSCLC models. The line within each notch box represents the median, and the lower and upper boundaries of the box indicate first and third quartiles, respectively. Error bars (whiskers) represent the 1.5-fold interquartile range. The *P*-value was calculated by pairwise comparison of groups using the Student's *t-*test.
