# Supplementary material for: Setting up a wide panel of patient-derived tumor xenografts of non–small cell lung cancer by improving the preanalytical steps
Source: Cancer Med. 2014 Dec 3;4(2):201–11. doi: 10.1002/cam4.357 (PMC4329004; doi:10.1002/cam4.357)
Supplement: Supplementary file 1 [file cam40004-0201-sd1.pptx]

## Slide 1
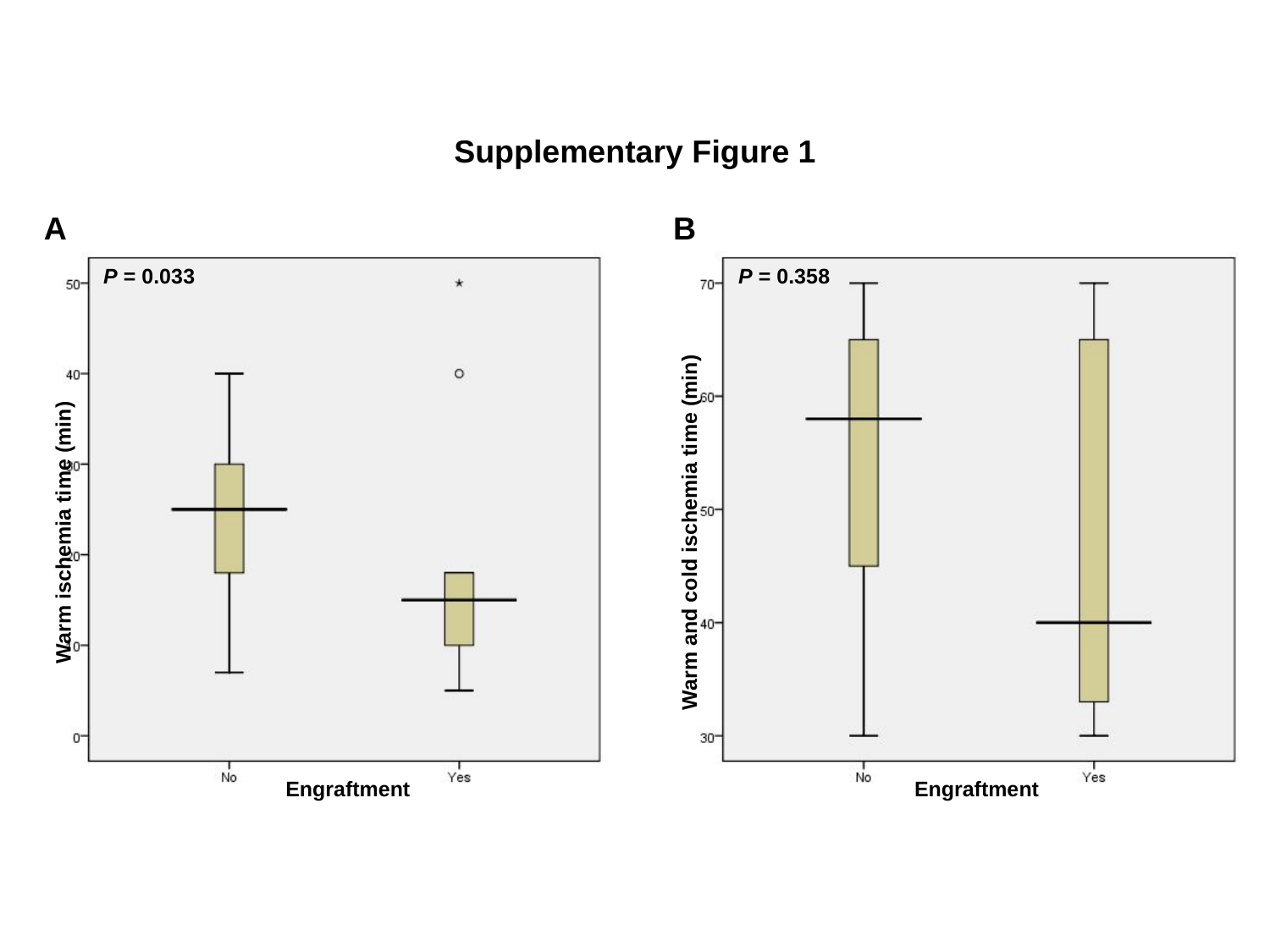

Supplementary Figure 1
A
B
P = 0.033
P = 0.358
Warm ischemia time (min)
Warm and cold ischemia time (min)
Engraftment
Engraftment
